# Dr. Pease’s Case Reported in the October Number

**Published:** 1895-12

**Authors:** F. O. Pease

**Affiliations:** 103 State Street, Chicago


### DR. PEASE'S CASE, REPORTED IN OCTOBER NUMBER.

I am pleased to say that our patient, Miss M. K., reported in the October number of The Homœopathic Physician, at page 458, is now a buxom miss of healthy and womanly appearance; has had no illness since last year, and has fully recovered the use of her limbs and mental powers; functions are normal, and she is rapidly developing into womanhood.

F. O. Pease, M. D.,
103 State Street, Chicago.

October 2d, 1895.

